# A Dual Inhibitor of Cdc7/Cdk9 Potently Suppresses T Cell Activation

**DOI:** 10.3389/fimmu.2019.01718

**Published:** 2019-07-25

**Authors:** Elijah W. Chen, Neil Q. Tay, Joanna Brzostek, Nicholas R. J. Gascoigne, Vasily Rybakin

**Affiliations:** ^1^Department of Microbiology and Immunology, Yong Loo Lin School of Medicine, National University of Singapore, Singapore, Singapore; ^2^Immunology Programme, Centre for Life Sciences, Life Sciences Institute, National University of Singapore, Singapore, Singapore; ^3^Centre for Life Sciences (CeLS), NUS Graduate School for Integrative Sciences and Engineering (NGS), National University of Singapore, Singapore, Singapore; ^4^Laboratory of Immunobiology, Rega Institute for Medical Research, KU Leuven, Leuven, Belgium

**Keywords:** Cdc7, Cdk9, PHA-767491, TCR signaling, T cell activation, thymocyte selection

## Abstract

T cell activation is mediated by signaling pathways originating from the T cell receptor (TCR). Propagation of signals downstream of the TCR involves a cascade of numerous kinases, some of which have yet to be identified. Through a screening strategy that we have previously introduced, PHA-767491, an inhibitor of the kinases Cdc7 and Cdk9, was identified to impede TCR signaling. PHA-767491 suppressed several T cell activation phenomena, including the expression of activation markers, proliferation, and effector functions. We also observed a defect in TCR signaling pathways upon PHA-767491 treatment. Inhibition of Cdc7/Cdk9 impairs T cell responses, which could potentially be detrimental for the immune response to tumors, and also compromises the ability to resist infections. The Cdc7/Cdk9 inhibitor is a strong candidate as a cancer therapeutic, but its effect on the immune system poses a problem for clinical applications.

## Introduction

Cell cycle progression is controlled by an intricate network of tightly regulated factors, key among which are cyclins and cyclin-dependent kinases (Cdk/Cdc) (1). Breakdown of cell cycle control mechanisms represents one of the keystones of cancer, and targeting cell cycle regulation is a recurring theme in cancer therapy (2, 3). A growing number of cell permeable small molecules interfering with mitotic checkpoints have entered clinical studies, either alone or with conventional chemotherapy, e.g., antimetabolites or microtubule inhibitors (4). For example, an inhibitor of the G2-M DNA damage checkpoint kinase Wee1 is undergoing multiple clinical trials as a monotherapy or combination therapy for several types of solid tumors (5), whereas spindle assembly checkpoint kinases Plk and Aurora A/C are targeted in trials in patients with pancreatic cancer, advanced non-small cell lung carcinoma, lymphoma, and other cancers (6, 7).

Cdc7 is an essential kinase required for the progression through cell cycle. Cdc7 forms an active kinase complex upon association with its cofactor Dbf4 (8–10). This complex phosphorylates several components of the DNA helicase machinery, notably the mini-chromosome maintenance protein MCM2 (11), thereby activating the helicase activity and permitting DNA unwinding. Thus, Cdc7 plays a critical regulatory role in the firing of origins of replication, facilitating the process of DNA replication and driving the transition from G1 to S phase of the cell cycle. Deletion of Cdc7 is lethal early in embryogenesis (12). Cdc7 is overexpressed in a number of cancers and may represent a prognostic marker (13). Notably, the overexpression of the kinase exerts direct protective effects. Experiments in tissue culture demonstrated that SCC-9 and SCC-15 squamous cell carcinoma cells become progressively more resistant to apoptosis induction by ultraviolet light and chemical agents such as hydroxyurea and camptothecin with the increase of Cdc7 expression (14). This effect may be mediated by the loss of p53 (15). Analysis of human breast cancer samples revealed a positive correlation between Cdc7 expression and proliferative activity and a number of gene amplifications (16).

The unique position of Cdc7 at the initiation of replication offered exciting prospects for pharmacological interference. An early report on the therapeutic effect of Cdc7 inhibition suggested that chemical interference with Cdc7 may predominantly target cancer cells (17). Based on this finding, a number of animal models of cancer were investigated, yielding highly promising results. The compound PHA-767491 (1,5,6,7-Tetrahydro-2-(4-pyridinyl)-4H-pyrrolo[3,2-c]pyridin-4-one hydrochloride), an ATP-competitive inhibitor of Cdc7 (18), displayed strong tumor suppression in several mouse models and good drug tolerance following intravenous application with doses up to 30 mg/kg (18). A chemically distinct compound XL-413 (8-Chloro-2-(2S)-2-pyrrolidinylbenzofuro[3,2-d]pyrimidin-4(3H)-one hydrochloride) displayed strong anticancer activity against Colo-205 xenografts in mice and good drug tolerance to oral doses up to 100 mg/kg (19). Both compounds were highly toxic to cancer cells *in vitro*. Several phase I and I/II clinical studies were initiated to evaluate the safety and efficacy of PHA-767491 and XL-413 in human patients. In the US, four clinical trials of Cdc7 inhibitors BMS-863233/XL-413, and NMS-1116354/PHA-767491 in advanced solid tumors and hematologic cancers were terminated (www.clinicaltrials.gov, trials NCT00838890, NCT00886782, NCT01016327, and NCT01092052), while one phase I study is ongoing with a new compound, TAK-931 (US Trial NCT02699749). In the EU, a phase I study of XL-413 in advanced and/or metastatic solid tumors yielded devastating results (100% adverse effects incl. 54.5% severe; 27.3% death incl. 18.2% within 30 days of the completion of drug regimen; see EU Final Clinical Study Report CA198002, www.clinicaltrialsregister.eu). As detailed results have not been reported, the cause for the trial's failures is not publicly known. Nonetheless, the summarized list of laboratory abnormalities; low hemoglobin, low lymphocyte count, low white blood cell count, low neutrophil count, suggests an impairment of the immune system.

Despite its intended and reported use in pre-clinical research and clinical trials to inhibit Cdc7, the compound PHA-767491 co-targets Cdk9, a protein kinase which is not directly related to cell cycle control, but rather regulates global transcription (20). The reported IC_50_ values are 10 nM for Cdc7 and 34 nM for Cdk9 (21). Cdk9 in its own right has been considered a promising target in cancer therapy (22). However, similarly to Cdc7 above, several early stage trials demonstrated severe adverse effects, as summarized in (23). Several different drugs have been trialed as Cdk9 inhibitors, with Flavopiridol/Alvocidib being the most widely tested in several phase I and phase II studies (NCT00082784, NCT00083122, NCT00112723, NCT00407966, NCT00464633, NCT00470197, and NCT00735930). Generally, the effectiveness of Flavopiridol varies across the different studies, but several adverse events types were frequently reported, including immune system related adverse events such as neutropenia, various infections, and edema (23). However, Flavopiridol/Alvocidib was approved by the FDA as an orphan drug for acute myeloid leukemia. Dinaciclib/SCH-727965 is another Cdk9 inhibitor with several phase I and II trials to its name (NCT00732810, NCT00798213, NCT00871663, NCT01096342, and NCT01650727). Similarly, several immune related adverse effects such as neutropenia and leukopenia were reported. It was also given an orphan drug status for chronic lymphocytic leukemia. Other inhibitors to have entered clinical studies include Roscovitine/Seliciclib/CYC202, SNS-032, RGB-286638, BAY-1251152, and TG02. The comparatively low selectivity of Cdc7 and Cdk9 inhibitors is considered as one of the probable reasons for the unsuccessful translation from animal models to human trials (23). Nonetheless, Cdk9-targeted therapy is still very much in vogue. Current developments include an oral version of alvocidib in ongoing Phase I trials (NCT03604783), and AZD4573, a Cdk9 inhibitor administered via IV, is also undergoing Phase I trials (NCT03263637). To the best of our knowledge, inhibition of Cdk9 by XL-413 has not been reported.

In this study, we investigated Cdc7/Cdk9 inhibitor PHA-767491 as a hit from a small molecule screen designed to identify new modulators of T cell development and activation (24, 25). We show that PHA-767491 and other Cdc7 and Cdk9 inhibitors potently suppress T cell activation, proliferation, and effector functions. In addition to effects that can be directly attributed to the inhibition of the DNA helicase activity and RNA polymerase II, we identified several unexpected effects of Cdc7/Cdk9 inhibition early in T cell receptor signaling, suggesting that Cdc7 or Cdk9 may play a previously undescribed role in TCR signal transduction. Our data indicate that systemic interference with Cdc7/Cdk9 activity results in a strong suppression of the adaptive immune response and likely affects not only the adaptive response to tumors but also the ability of the immune system to react to cancer-associated opportunistic infections (26).

## Materials and Methods

### Mice

C57BL/6J (B6; cat. #000664) and OT-I transgenic (C57BL/6-Tg(TcraTcrb)1100Mjb/J; cat. #003831) mice were used at between 6 and 8 weeks of age. The strains were obtained from the Jackson Laboratory, and were later bred in the National University of Singapore Comparative Medicine animal facility under restricted flora conditions in accordance with National University of Singapore IACUC guidelines.

### Cells and Inhibitors

Thymocytes and lymphocytes were taken from B6 mice and kept in RPMI media supplemented with 10% FCS, 100 U/ml penicillin, 0.1 mg/ml streptomycin, 2 mM L-glutamate, 50 μM β-mercaptoethanol (β-ME), and 1 mM sodium pyruvate. Jurkat cells (clone E6.1) were cultured in RPMI medium containing 10% FCS, 2 mM L-glutamate, 50 μM β-ME, and 1 mM sodium pyruvate.

OT-I CTL were prepared by culturing splenocytes in RPMI supplemented with 10% FCS, 100 U/ml penicillin, 0.1 mg/ml streptomycin, 2 mM L-glutamate, 50 μM β-ME, 1 mM sodium pyruvate, 10 mM HEPES, 0.1 mM NEAA. 10 U/ml IL-2 and 10 nM OVA peptide were added to the media for the first 2 days of culture, after which the cells were resuspended in cRPMI without the OVA peptide for another 2 days.

Human PBMCs were cultured in RPMI medium containing 10% FCS, 2 mM L-glutamate, 50 μM β-ME, and 1 mM sodium pyruvate. PBMCs were collected from healthy volunteers under a National University of Singapore IRB-approved protocol. Informed consent was obtained from all volunteers.

Stimulations of cells with anti-CD3/CD28 magnetic beads were carried out at a ratio of 1 bead to 2.5 cells. For plate-bound stimulations, purified anti-CD3 antibody was plated at 2 μg/ml. Stimulations using tetramers were conducted in a 37°C circulating water bath with 1 μl of tetramers added for every 1 × 10^6^ cells. In cell-mediated stimulations, APCs were plated 1 day prior and co-cultured with T cells at a ratio of 1 APC to 10 T cells, unless otherwise stated. PMA and ionomycin were added to cells at 100 ng/ml and 1 μM, respectively.

Kinase inhibitors were obtained from a kinase inhibitor screening library by Cayman Chemical (#10505). PHA-767491, 2-(pyridin-4-yl)-6,7-dihydro-1H-pyrrolo[3,2-c]pyridin-4(5H)-one hydrochloride, was obtained from Sigma. XL413, (S)-8-chloro-2-(pyrrolidin-2-yl)benzofuro[3,2-d]pyrimidin-4(3H)-one hydrochloride, was obtained from BioVision. SNS-032, N-(5-((5-tert-butyloxazol-2-yl)methylthio)thiazol-2-yl)piperidine-4-carboxamide, and LDC000067, 3-[[6-(2-methoxyphenyl)-4-pyrimidinyl]amino]-benzenemethanesulfonamide, were both obtained from Selleck Chemicals. zVAD-FMK was purchased from R&D Systems.

### Antibodies and Reagents

The antibodies used in this study for flow cytometry include: anti-mouse CD8 (#563786), anti-mouse CD25 (#558642), anti-mouse TCRβ (#553174), anti-human CD3 (#563798), anti-human CD8 (#563795), and anti-human TCRαβ (#563826) from BD Biosciences; anti-mouse CD4 (#100544), anti-mouse IFN-γ (#505835), anti-human LFA-1 (#363404), anti-human CD11a (#301208), anti-human CD18 (#302114), anti-human CD25 (#302625), and anti-human CD69 (#310920) from BioLegend; anti-mouse CD4 (#17-0042-83), anti-mouse CD4 (#17-0041-83), anti-mouse CD69 (#25-0691-82), anti-mouse TNF (#17-7321-82), anti-mouse IL-2 (#12-7021-82), anti-human CD4 (#17-0048-2), and anti-human CD8 (#11-0088-41) from eBioscience.

The antibodies used for immunoprecipitation and immunoblotting include: anti-Cdc7 (#ab10535), anti-Dbf4 (#ab124707), anti-RNA polymerase II (#ab817) and anti-RNA polymerase II (phospho S2, # ab193468) from Abcam; anti-PLCγ (#610027) and anti-ERK (#610031) from BD Biosciences; anti-LAT (#641102) from BioLegend; anti-GAPDH (#2118), anti-pPLCγ (#2821), anti-ZAP70 (#2709), anti-pZAP70 (#2717), anti-pERK (#4370), anti-pLAT (#3584), anti-pLck (#6943), and anti-NF-κB (#3035) from Cell Signaling; anti pMCM2 S40 (#GTX62847) from GeneTex; anti-MCM2 (#MA5-15895) from Pierce; and anti-Lck (#sc-433) from Santa Cruz.

### Flow Cytometric Analysis

Active caspase-3 staining was carried out using an active caspase-3 staining kit from BD Pharmingen (#550480). The staining procedures were carried out as detailed previously (24, 25).

For stimulation assays around 3 h, cells were stained with surface antibodies for CD4, CD8, CD69, and TCRβ. For stimulation assays lasting between 17 and 24 h, cells were stained with surface antibodies for CD4, CD8, CD25, CD69, and TCRβ. For proliferation assays, cells were first stained with proliferation marker CellTrace Violet (CTV) prior to incubation and stained with surface antibodies CD4, CD8, and CD25 prior to analysis. For cytokine assays, cells were stimulated with APCs, K^b^-OVA tetramers, or PMA and ionomycin, stained with surface antibodies for CD4 and CD8, fixed and permeabilized, and stained for the intracellular cytokines IFN-γ, TNF, and IL-2. For pErk staining, cells were stimulated with K^b^-OVA tetramers conjugated with PE. The cells were fixed with an equal volume of 8% PFA after stimulation and permeabilized with methanol and then stained for pErk and CD8.

Cells were acquired with the BD LSRFortessa X-20 flow cytometer (BD Biosciences) and analyzed using the FlowJo (Treestar) software.

### LFA-1 Activation Assay

Human PBMCs were pre-treated with DMSO or PHA-767491 for 15 min at 37°C. 1 × 10^6^ PBMCs were seeded into each well of a 24-well plate and stimulated with either anti-CD3/CD28 beads, at a ratio of 2.5 cells per bead, or 50 ng/ml PMA. The cells were stimulated at 37°C for up to 3 h, along with the addition of the LFA-1 antibody. After stimulation, the cells were surface stained for CD4, CD8, CD11a, and CD18. The samples were acquired using the BD LSRFortessa X-20 flow cytometer.

### Cell Viability Assays

CTL were cultured from OT-I splenocytes grown as described above. EL4 cells were first stained with 5 μM CTV, pulsed with OVA peptide, and co-cultured with the OT-I CTL. EL4 cells were seeded at 2 × 10^5^ cells/well, and effector to target cell ratios ranged from 1:1 to 8:1. The cells were treated with DMSO or PHA-767491 and incubated at 37°C, 5% CO_2_ for 5 h. The cells were stained for surface CD8, along with a live/dead dye and resazurin (Thermo Fisher Scientific), a viability dye.

### Immunoprecipitation and Immunoblotting

For immunoprecipitation, 10–20 × 10^6^ Jurkat cells were lysed with RIPA lysis buffer (Thermo Fisher Scientific) supplemented with HALT™ protease and phosphatase inhibitor (Thermo Fisher Scientific). Lysed cells were centrifuged at 14,000 rpm, 15 min, 4°C and subsequently resuspended in primary antibodies (mentioned above) and incubated at 4°C overnight. Protein G magnetic beads (Invitrogen) were added and incubated at 4°C for 3 h. The magnetic beads were separated from the supernatant and resuspended in 1X sample buffer (Nacalai Tesque).

For immunoblotting assays, 5 × 10^6^ CTL or 2 × 10^6^ Jurkat cells were lysed with RIPA lysis buffer supplemented with HALT™ protease and phosphatase inhibitor cocktail. Samples were ran on SDS-PAGE, transferred to PVDF membranes and were probed with specific primary antibodies (mentioned above) at 4°C overnight. Secondary antibodies used include goat anti-rabbit IgG, 680LT (LI-COR Biosciences, #926-68021) or goat anti-mouse IgG, 800CW (LI-COR Biosciences, #926-32210). Membranes were scanned and quantified with LiCor Odyssey infrared imaging system (LI-COR Biosciences).

### Calcium Flux

Lymphocytes were prepared at 1 × 10^7^ cells/ml in cRPMI and treated with 2 μM Indo-1-AM and incubated for 30 min at 37°C and 5% CO_2_. Biotinylated anti-CD3 were added to the cells and left on ice for 30 min. Surface staining of CD4 and CD8 was also done concurrently. Samples were resuspended in Ca^2+^ and Mg^2+^ free cHBSS. Samples were analyzed on BD LSRFortessa X-20 while being kept warm at around 37°C. Streptavidin was added at 2 μg/ml to induce TCR cross-linking. CaCl_2_ was added at 5 mM to induce SOCE. Ionomycin was added at 1 μM to induce maximal Ca^2+^ flux.

### Luciferase Quantitation Assays

Jurkat cells carrying a reporter for NFAT activity were generated by the Acuto lab, as documented previously (27). The NFAT reporter cells were stimulated with plate-bound anti-CD3 antibodies. The assay was carried out according to the manufacturer's protocol (Promega, #E1500).

Jurkat cells were electroporated with NF-κB firefly luciferase construct (Promega #E8491) and *Renilla* luciferase construct using ECM 830 BTX electroporation system (BTX). Cells were cultured with hygromycin selection media for 1 day. Selected Jurkat cells were stimulated with plate-bound anti-CD3 antibodies. Luciferase activity was measured for firefly luciferase and *Renilla* luciferase activity in sequence, as per the manufacturer's protocol (Promega, #E2920). Luciferase activity was measured using the infinite M200 plate reader (Tecan).

## Results

### Kinase Inhibitor Library Screen Reveals a Cdc7/Cdk9 Inhibitor as a Modulator of T Cell Activation

**Figure 1:**
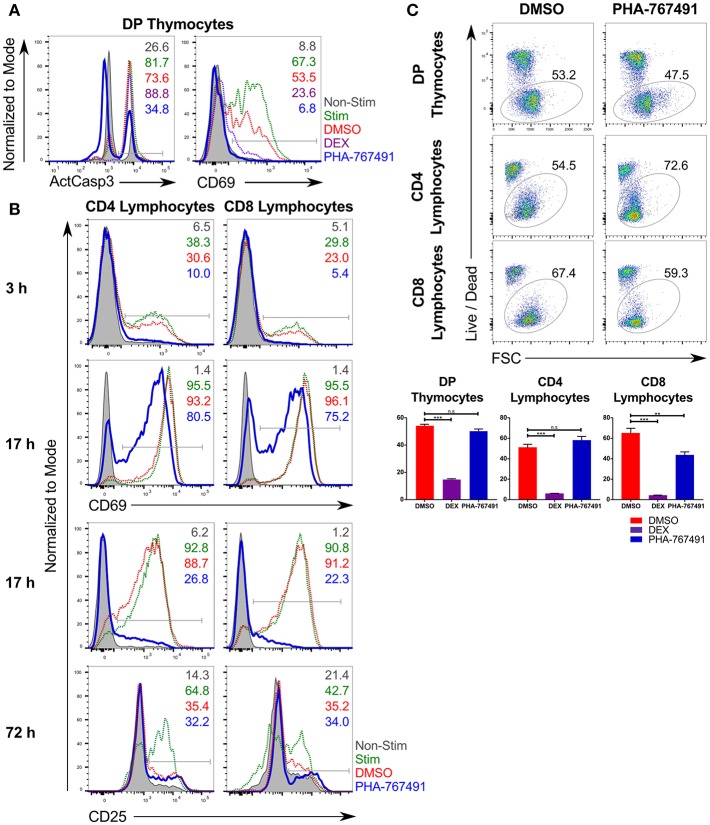
PHA-767491 suppresses T cell activation. **(A)** PHA-767491 suppresses activation of caspase-3 and expression of CD69 in mouse thymocytes stimulated with anti-CD3/CD28 beads for 17 h. **(B)** PHA-767491 suppresses expression of CD69 and CD25 in mouse peripheral T lymphocytes stimulated with plate-bound anti-CD3 antibodies for 3, 17, or 72 h. **(C)** PHA-767491 does not affect the viability of primary mouse thymocytes or stimulated peripheral T lymphocytes after 17 h incubation. Cells were incubated with 1% DMSO (DMSO; negative vehicle control), 5 μM dexamethasone (DEX; positive control), or 10 μM PHA-767491. Viable cells were detected based on Live/Dead staining. Data shown is representative of at least three independent experiments. Bar charts are represented as mean ± SEM. Statistical significance was determined by unpaired two-sided Student's *t*-test (n.s, non-significant; **p < 0.01; ***p < 0.001). The percentages of the positive population of each sample are represented in each graph according to their respective colors. ActCasp3, active caspase-3; non-stim, non-stimulated; stim: stimulated; DMSO, stimulated vehicle controls treated with DMSO; DEX, treated with dexamethasone.

We have recently reported a method of small molecule library screening designed to identify potential new targets in the TCR signaling pathway (24, 25). CD3/CD28 stimulation induces strong TCR signaling in primary thymocytes, resulting in caspase-3 activation and cell death, mimicking negative selection (28). We screened a library of kinase inhibitors for their effect on suppression of apoptosis in C57BL/6 mouse thymocytes stimulated with anti-CD3/CD28 magnetic beads. Among the most potent hits was the compound PHA-767491, a previously characterized inhibitor of the protein kinases Cdc7 and Cdk9 (Figure 1A). Cdc7 is a serine/threonine kinase that phosphorylates and activates members of the DNA helicase complex, thus allowing the progression of the cell cycle through the S phase (8, 29). Cdk9 is a kinase that phosphorylates RNA polymerase II (RNAPII) and facilitates transcription (30, 31). A separate series of screens were undertaken to assess the inhibitor's effect on peripheral T cell activation. These experiments revealed that PHA-767491 also suppressed the activation of lymphocytes, as indicated by the delayed onset of CD69 and CD25 expression (Figure 1B). Importantly, even prolonged incubation with the compound in the absence of antigenic stimulation did not result in decreased cell viability in primary mouse thymocytes (Figure 1C). In peripheral T cells, stimulated CD8 T cells show a higher susceptibility to PHA-767491-mediated toxicity compared to their CD4 counterparts (Figure 1C).

To formulate a range of working concentrations for PHA-767491, we examined the effect of different concentrations of PHA-767491 on lymphocytes from C57BL/6 mice stimulated by plate-bound anti-CD3 antibodies. Incubation with PHA-767491 suppressed CD25 expression on the stimulated lymphocytes in a dose-dependent manner (Figure S1A). The working range of PHA-767491 is in agreement with earlier studies, where concentrations of the compound ranging from 10 to 0.1 μM were found to inhibit cell proliferation with reported IC_50_ values around 0.6 μM (18, 32, 33). The expression of activation markers was inhibited by PHA-767491 in a dose dependent manner in both thymocytes and peripheral lymphocytes (Figure S1B).

### Inhibition of Canonical Cdc7 and Cdk9 Functions in PHA-767491-Treated T Cells

We sought to confirm that the application of PHA-767491 indeed suppressed the activities of its main targets, the protein kinases Cdc7 and Cdk9. MCM2, a member of the MCM2-7 helicase complex, is an established target of Cdc7, and is phosphorylated by Cdc7 on serine residues 40 and 53 (18, 34). Previous studies have shown that phosphorylation of MCM2 is reduced over time in response to Cdc7 inhibition (18, 34), thereby linking the phosphorylation of MCM2 to Cdc7 activity. MCM2 phosphorylation is used as a reliable indication of Cdc7 activity in pharmacological studies (19). Indeed, the amount of phosphorylation of MCM2 from unstimulated Jurkat cells and other cell lines decreased in response to PHA-767491 treatment (Figure S2), reflecting reduced Cdc7 activity.

**Figure 2:**
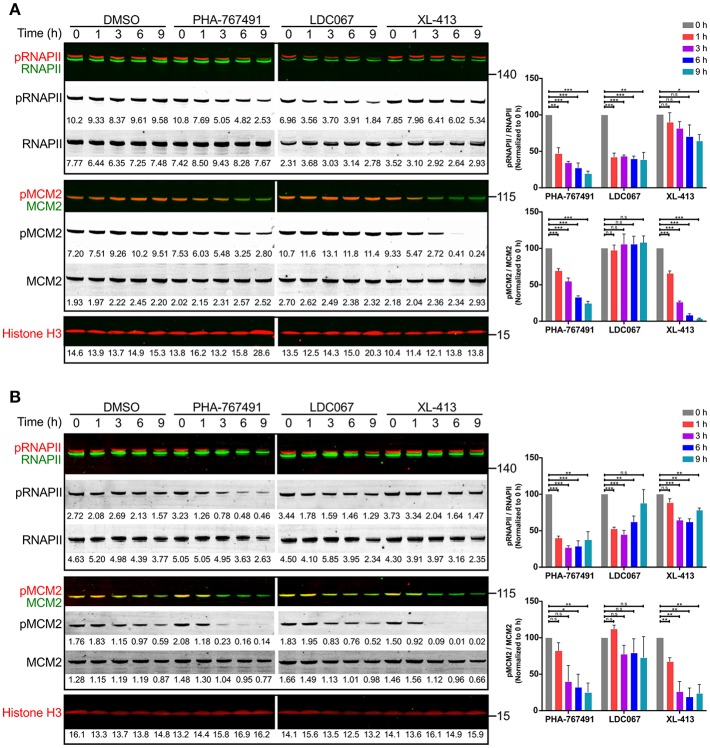
PHA-767491 inhibits the function of canonical targets Cdc7 and Cdk9. PHA-767491 inhibits the phosphorylation of both RNAPII (S2) and MCM2 (S40) in **(A)** Jurkat cells and **(B)** OT-I CTL. The cells were incubated with the respective inhibitors for the indicated duration. Raw values for the band intensities are shown below the respective bands. Representative blots of three independent experiments are shown. The averaged values of the ratio of the band intensities for both RNAPII and MCM2, normalized to their respective 0 h samples, are shown in bar charts to the right. Bar charts are represented as mean ± SEM. Statistical significance was determined by unpaired two-sided Student's *t*-test (n.s, non-significant; *p < 0.05; **p < 0.01; ***p < 0.001).

The other target of PHA-767491, Cdk9, phosphorylates RNAPII on the serine residue 2 of the carboxy-terminal domain (CTD) (23, 35). Inhibition of Cdk9 interferes with the phosphorylation of Ser2 of the CTD of RNAPII, resulting in a blockade in the elongation step of transcription. We proceeded to test the effect of PHA-767491 on the inhibition of Cdk9 through measuring phosphorylation of RNAPII in Jurkat cells and OT-I CTL that were treated with PHA-767491. In addition, we included a Cdk9-specific inhibitor, LDC000067 (henceforth abbreviated to LDC067) (35), and a Cdk7-specific inhibitor, XL-413 (19), for comparison. As expected, the dual kinase inhibitor PHA-767491 suppressed phosphorylation of both RNAPII and MCM2 in both Jurkat cells (Figure 2A) and OT-I CTL (Figure 2B). As a side note, the amount of Cdc7 declined over time upon PHA-767491 treatment, albeit at a slower rate than the loss of phosphorylation in MCM2 (Figure S2). Our results also confirmed the specificity of LDC067 and XL-413 for Cdk9 and Cdc7, respectively (Figure 2). LDC067-mediated inhibition of RNAPII appears relatively stable over time, which was also reported by Albert et al. (35), compared to PHA-767491, which showed a trend of decreasing RNAPII phosphorylation over time (Figure 2A). PHA-767491 also showed a stronger inhibitory effect on RNAPII phosphorylation than LDC067. In contrast, both PHA-767491 and XL-413 exerted increasing inhibitory effects on MCM2 phosphorylation over time (Figure 2), with XL-413 exhibiting stronger inhibition of phosphorylation than PHA-767491.

As the amount of phosphorylation of MCM2 proved to be a reliable indicator of Cdc7 activity, we proceeded to investigate the kinetics of PHA-767491-mediated inhibition of MCM2 phosphorylation. We stimulated Jurkat cells with PMA for different durations and measured the phosphorylation of MCM2. Inhibition of Cdc7 at early time points reduced the phosphorylation of MCM2 (Figure S3), indicating that Cdc7-mediated phosphorylation of MCM2 Ser40 can be modulated at early time points upon Cdc7 inhibition.

### Suppression of T Cell Proliferation and Effector Functions in PHA-767491-Treated T Cells

**Figure 3:**
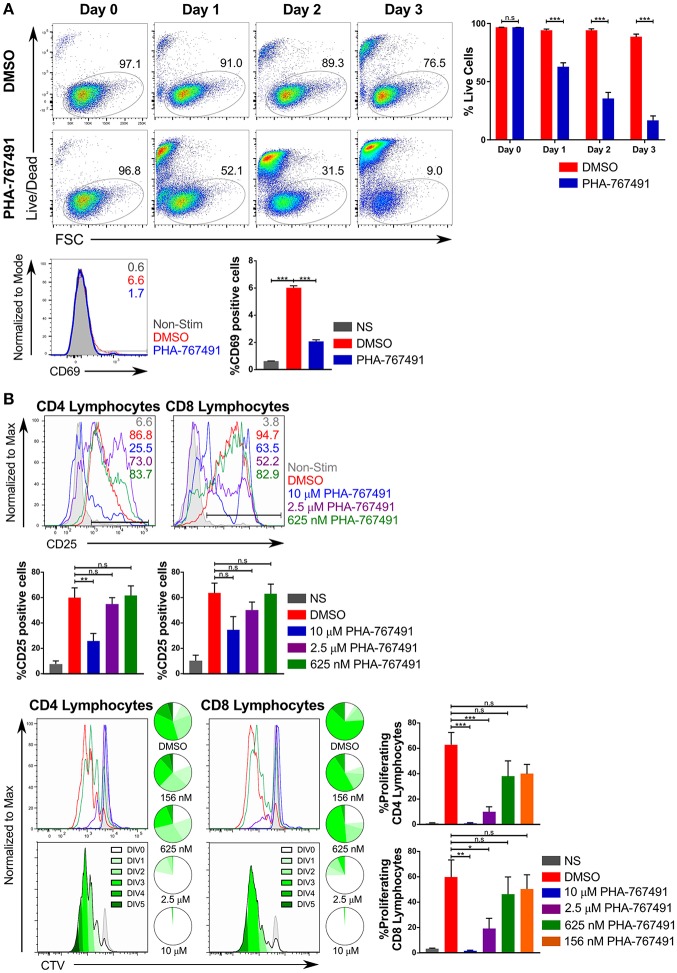
PHA-767491 impedes cell proliferation. **(A)** (Top) PHA-767491 suppresses constitutive proliferation and activation of Jurkat cells. Jurkat cells that were not TCR stimulated were treated with either DMSO or PHA-767491 and incubated for 72 h. A time course of Jurkat cell viability tracks the percentage of live Jurkat cells based on Live/Dead staining. (Bottom) Jurkat cells were stimulated with anti-CD3/CD28 antibody-coated beads for 3 h. The percentages of the positive population of each sample are represented in each graph according to their respective colors. **(B)** PHA-767491 suppresses proliferation of stimulated mouse lymphocytes. Peripheral mouse lymphocytes were labeled with CTV and subsequently treated with either DMSO or PHA-767491, seeded onto plates coated with plate-bound anti-CD3 antibodies and incubated for 72 h. The percentages of the positive population of each sample are represented in each graph according to their respective colors. Cell proliferation was assessed by quantifying CTV dilution. Pie charts show the proportion of cells in each peak of division. A darker shade of green is used for each subsequent round of division. Top panels show the CTV graphs of the different samples in their respective colors. Bottom panels use the trace of the DMSO-treated sample to illustrate the individual division peaks and the corresponding colors. Data shown is representative of at least three independent experiments. Bar charts are represented as mean ± SEM. Statistical significance was determined by unpaired two-sided Student's *t*-test (n.s, non-significant; *p < 0.05; **p < 0.01; ***p < 0.001).

Because of the expected antiproliferative effect of Cdc7 inhibition, we examined the consequences of PHA-767491 treatment for T cell proliferation. PHA-767491-treated, unstimulated Jurkat cells exhibited a high proportion of dead cells, which was apparent within 24 h of the inhibitor treatment (Figure 3A). PHA-767491-treatment of Jurkat cells stimulated with plate-bound anti-CD3 antibody also showed inhibition of CD69 upregulation (Figure 3A). Suppression of proliferative responses was also evident in *ex vivo* murine lymphocytes stimulated with plate-bound anti-CD3 antibodies (Figure 3B), which is in agreement with the canonical function of Cdc7 in the regulation of the cell cycle (29, 36).

**Figure 4:**
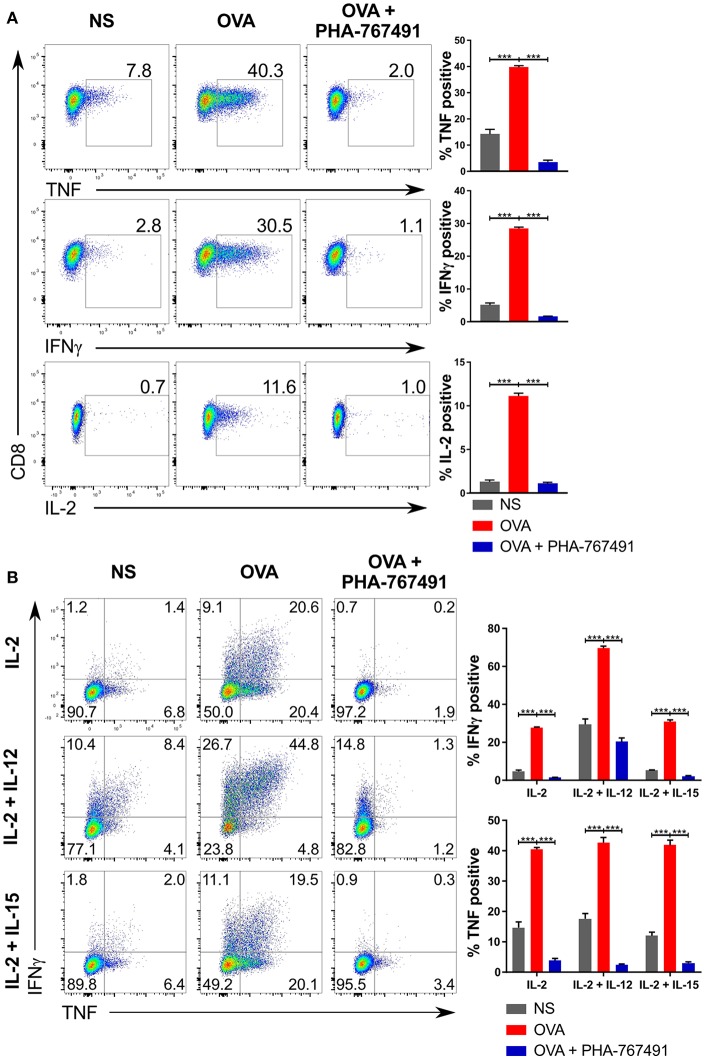
Suppression of cytokine production by PHA-767491. **(A)** PHA-767491 suppresses cytokine production in OT-I CTL. APCs expressing single chain H-2K^b^ with OVA peptide were seeded into 96 well plates 1 day prior to the experiment. CTL were pre-treated with BFA for 30 min before co-culturing with the APCs for 6 h. The percentages of the positive population of each sample are indicated for each plot. **(B)** PHA-767491 also suppresses cytokine production in strongly polarized OT-I CTL. For the preparation of the OT-I CTL, OT-I splenocytes were pre-treated with either 10 nM OVA peptide and 10 U/ml IL-2 only, 10 nM OVA peptide and 10 U/ml IL-2 with 10 ng/ml IL-12, or 10 nM OVA peptide and 10 U/ml IL-2 with 10 ng/ml IL-15. CTL were pre-treated with BFA and restimulated by co-culturing with APCs expressing single chain H-2K^b^ with OVA peptide for 6 h. The percentages of each quadrant in each graph are shown. Data shown is representative of at least three independent experiments. Bar charts are represented as mean ± SEM. Statistical significance was determined by unpaired two-sided Student's *t*-test (***p < 0.001).

Because of the expected suppression of gene expression due to the inhibition of Cdk9, we assessed the effect of PHA-767491 on the production of effector cytokines in OT-I CTL stimulated with CHO cells expressing single-chain trimer of H-2K^b^ presenting the antigenic SIINFEKL (OVA) peptide (37). Addition of the inhibitor suppressed the production of TNF, IL-2 and IFN-γ (Figure 4A).

To further validate the suppression of cytokine production by the inhibitor, we treated *ex vivo* OT-I murine peripheral lymphocytes stimulated with K^b^-OVA tetramers. In agreement with the OT-I CTL results, stimulation of the peripheral lymphocytes led to cytokine production, which was inhibited by PHA-767491 (Figure S4A). Although the cytokine production by stimulated peripheral T cells is at a much lower level compared to the CTL, it is in line with reports that *ex vivo* lymphocytes are less capable cytokine producers than effector or memory T cells (38, 39). Nonetheless, the inhibition of cytokine production in naïve OT-I cells replicates the observations made with OT-I CTL. Likewise, *ex vivo* B6 wild-type peripheral lymphocytes stimulated by PMA and ionomycin also showed reduced cytokine production (Figure S4A). Furthermore, using the OT-I peripheral lymphocytes stimulated with K^b^-OVA tetramers, we were also able to replicate earlier results including the inhibition of activation markers in a 3 h stimulation assay (Figure S4B), and the suppression of proliferation (Figure S4C).

The near complete suppression of IFN-γ led us to test the extent of inhibition of IFN-γ production in OT-I CTL subjected to stronger forms of stimulation. We pre-activated OT-I splenocytes with only OVA and IL-2 or supplemented with either IL-12 or IL-15 for a period of 4 days, followed by a re-stimulation with H-2K^b^-OVA expressing APCs, with or without addition of PHA-767491. Again, production of characteristic cytokines TNF and IFN-γ was strongly suppressed by Cdc7/Cdk9 inhibition (Figure 4B).

**Figure 5:**
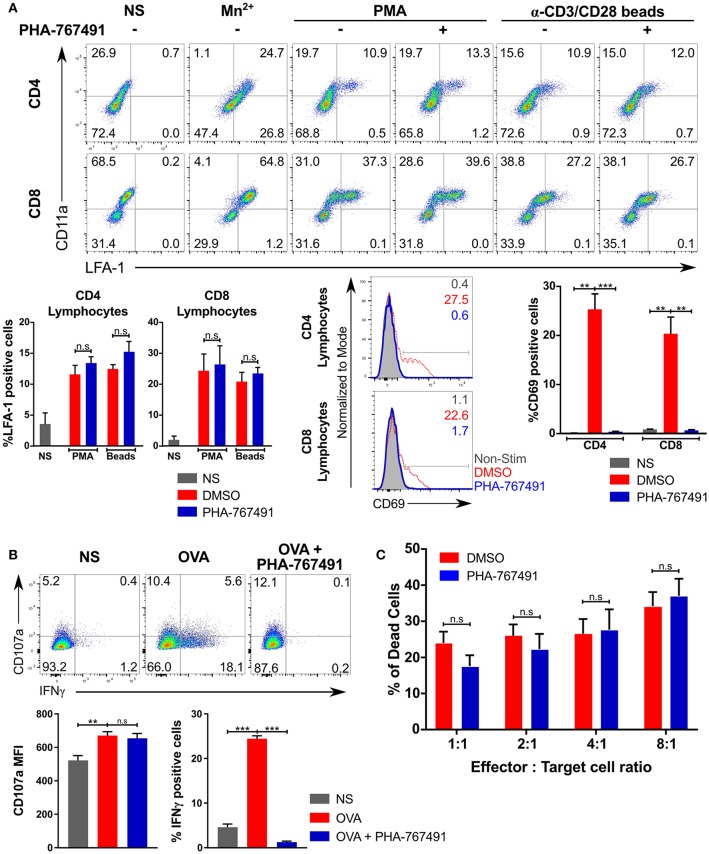
Effector functions not inhibited by PHA-767491. **(A)** PHA-767491 does not block LFA-1 conformational change. Human PBMCs were stimulated with PMA or anti-human CD3/CD28 beads for 0.5 and 3 h, respectively, and incubated with anti-LFA-1 antibody during the stimulation period. Mn^2+^ was used as a positive control for LFA-1 activation. The percentages of each quadrant in each graph are shown. PHA-767491 suppresses expression of CD69 in human T lymphocytes that were stimulated with anti-human CD3/CD28 beads for 3 h. **(B)** PHA-767491 does not impair degranulation in OT-I CTL. CTL were pre-treated with BFA for 30 min and stained with anti-CD107a antibody during the stimulation with APCs expressing single chain H-2K^b^ with OVA peptide for 6 h. The percentages of each quadrant in each graph are shown. **(C)** PHA-767491 does not dampen cytotoxicity of OT-I CTL. CTL that were serum-starved overnight were treated with either DMSO or PHA-767491 for 30 min before co-culturing with CTV-labeled and OVA-pulsed EL4 cells for 6 h. The bar charts compare the percentage of dead EL4 target cells, which were gated based on positive staining for the Live/Dead stain and negative staining for resazurin. Data shown is representative of at least three independent experiments. Bar charts are represented as mean ± SEM. Statistical significance was determined by unpaired two-sided Student's *t*-test (n.s, non-significant; **p < 0.01; ***p < 0.001).

Several effector functions were unaffected by the inhibitor. The integrin signaling pathway represents one of the early T cell activation phenomena, and the pathway diverges early on from the other established branches that lead to transcription and protein synthesis (40). To determine the effects of Cdc7/Cdk9 inhibition on integrin activation, we stimulated human PBMCs with anti-CD3/CD28 beads or PMA and stained the cells for activated LFA-1, using the anti-LFA-1 (clone m24) antibody, which recognizes an epitope on activated human LFA-1 in the activated conformation. There was no apparent inhibition of the activation of LFA-1 with PHA-767491 treatment, while the overall expression of the integrin, based on CD11a staining, was constant (Figure 5A). This lack of inhibition of the LFA-1 “inside-out” signaling is not attributed to a lack of inhibition in human T cells as CD69 upregulation was clearly inhibited by PHA-767491 (Figure 5A).

Degranulation is a canonical effector cytolytic function in CTL. In CTL stimulated with K^b^-OVA expressing APCs, degranulation did not seem to be impaired by the drug as the surface expression of CD107a (LAMP1) was not affected (Figure 5B). In line with these findings, we found no effect of Cdc7/Cdk9 inhibition in cytotoxicity assays with EL4 target cells that were pulsed with the OVA peptide. EL4 cells were suitable for the assessment of cytotoxicity as it facilitates the use of a flow cytometry-based method to ascertain the effects of the inhibitor on the cytotoxic effects of OT-I CTL: a combination staining strategy of a live/dead stain with resazurin was employed to identify a metabolically active population and a dead cell population. The results indicated that PHA-767491 treatment did not suppress cytotoxicity at any combination of effector to target cell ratios tested (Figure 5C).

### Effects of Other Cdc7 and Cdk9 Inhibitors on T Cell Activation

We considered the possibility that our observations on the inhibition of T cell activation were merely due to a side effect of inhibiting the cell cycle. We compared the thymocyte library screening results of PHA-767491 to other cell cycle inhibitors. Unlike PHA-767491, cell cycle inhibitors such as NSC663284 (41) (inhibitor of Cdc25), Phthalazinonepyrazole (42) (inhibitor of Aurora A), ZM447439 (43) (inhibitor of Aurora B and C), (R)-Roscovitine (44) (pan-Cdk inhibitor, with strongest affinity for Cdk2/cyclin E), and Kenpaullone (45) (inhibitor of Cdk1/cyclin B and GSK3β) did not suppress the activation of caspase-3 or downregulate the expression of CD69 (Figure S5A). In particular, (R)-Roscovitine's ability to inhibit Cdk9 and Cdk2/cyclin E posits it as an inhibitor with similar effects as PHA-767491 in terms of inhibition of both transcription and cell cycle progression through G1 to S phase. Likewise, Kenpaullone's inhibition of Cdk1/cyclin B would impact exit from the S phase. Notably, in the original screen, the Cdk9/Cdk2 inhibitor CAY10574 (46) did not affect caspase-3 activity or CD69 upregulation (Figure S5A).

Nevertheless, we tested a set of compounds with different selectivities for Cdc7 vs. Cdk9. We compared the inhibitory effects of XL-413, a chemically distinct inhibitor of Cdc7 (19), to PHA-767491. In short-term stimulation assays, XL-413, like PHA-767491, suppressed the expression of CD69 in *ex vivo* mouse lymphocytes stimulated with plate-bound anti-CD3 antibodies (Figure S5B). Both XL-413 and PHA-767491 showed a similar dose-dependent effect on CD69 expression (Figure S5C).

**Figure 6:**
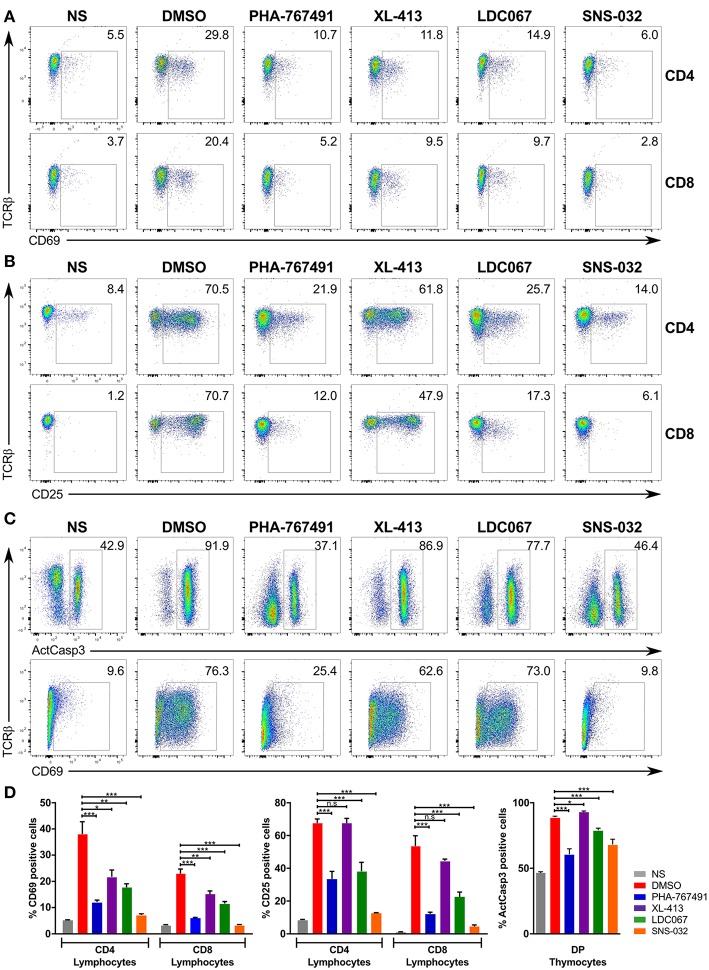
Effects of PHA-767491 inhibition can be reproduced by other Cdc7 and Cdk9 inhibitors. PHA-767491, XL-413, LDC067, and SNS-032 were used to examine the effects of Cdc7 and Cdk9 inhibition in **(A)** lymphocytes stimulated for 3 h, **(B)** lymphocytes stimulated for 17 h, or **(C)** thymocytes stimulated for 17 h. The percentages of the positive population of each sample are shown in the plots. Data shown is representative of at least three separate experiments. **(D)** The averaged results of the experiments from **(A–C)** are represented as mean ± SEM. Statistical significance was determined by unpaired two-sided Student's *t*-test (ns, non-significant; *p < 0.05; **p < 0.01; ***p < 0.001).

We conducted a series of stimulation assays to examine the effect of different Cdc7 and Cdk9 inhibitors on the response of T cells to stimulation. We added XL-413, LDC067, and SNS-032, which inhibits several Cdk proteins (47), to our inhibitor panel. The panel of inhibitors was applied to murine lymphocytes that were stimulated for 3 h (Figure 6A) or 17 h (Figure 6B) and to thymocytes that were stimulated for 17 h (Figure 6C). In terms of inhibition strength, SNS-032 showed the greatest inhibition of T cell activation marker expression, even exceeding that of PHA-767491 (Figure 6). LDC067 showed a similar inhibition profile to PHA-767491, except that it showed a weaker effect on thymocytes, both in the suppression of caspase-3 activation or CD69 expression (Figure 6). XL-413 suppressed CD69 upregulation in the short stimulation assays but exerted lower inhibitory potency in the longer stimulation assays (Figure 6). While the inhibition of activation marker expression by the Cdk9 inhibitors is an expected result due to the inhibition of transcription, the inhibition of T cell activation by XL-413 supports the notion that Cdc7 could be involved in the signaling events leading to T cell activation.

### Potential Involvement of Cdc7 in T Cell Receptor Signaling

**Figure 7:**
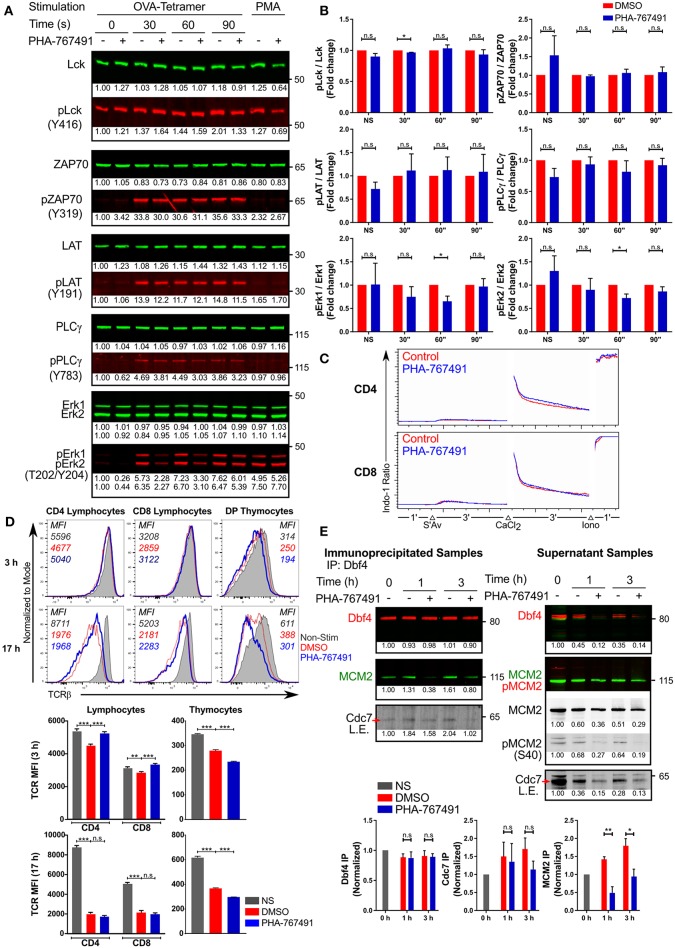
PHA-767491 modulates TCR signaling. **(A)** PHA-767491 treatment suppresses Erk phosphorylation. Immunoblots of OT-I CTL that were not-treated (–) or were treated (+) with PHA-767491 were stimulated with K^b^-OVA tetramers for the indicated times. Normalized values of individual bands are indicated below the respective bands. Representative blots of at least three independent experiments are shown. **(B)** The ratio of the band intensities of the phosphorylated proteins to the respective total proteins from **(A)** are shown in the bar charts, depicted as mean ± SEM. Values represent fold change, normalized to the DMSO-treated sample values for the respective time points. **(C)** PHA-767491 does not impair Ca^2+^ flux. Peripheral lymphocytes were either pre-treated with PHA-767491 or not and were incubated with biotinylated anti-CD3 antibodies. Lymphocytes were first cross-linked using streptavidin, followed by an exogenous addition of CaCl_2_ solution, and finally treated with ionomycin. **(D)** PHA-767491 affects TCR recycling in stimulation assays. Peripheral lymphocytes and thymocytes were stimulated with plate-bound anti-CD3 antibody or anti-CD3/CD28 beads, respectively, for 3 or 17 h. Values indicated in the histograms represent the MFI. **(E)** Cdc7 association with Dbf4 and MCM2 is reduced in TCR stimulated T cells treated with PHA-767491. Serum-starved Jurkat cells that were not-treated (–) or treated (+) with PHA-767491 were seeded into wells coated with plate-bound anti-CD3 antibodies and incubated for the indicated times and lysed. Anti-Dbf4 antibodies were used to immunoprecipitate Dbf4 from the cell lysates. Samples were immunoblotted for immunoprecipitated Dbf4 and co-immunoprecipitated Cdc7 and MCM2. Data shown is representative of at least three independent experiments. Bar charts are represented as mean ± SEM. Statistical significance was determined by unpaired two-sided Student's *t*-test (n.s, non-significant; *p < 0.05; **p < 0.01; ***p < 0.001).

We endeavored to investigate the potential involvement of Cdc7 in T cell receptor (TCR) signal transduction by analyzing the activation of several canonical molecules of the TCR signaling pathway. To ensure compatibility of the stimulation assay with a short stimulation timeframe, we employed the use of the transgenic OT-I CTL and stimulated them with the high affinity H-2K^b^-OVA tetramers. In order to identify the timepoint where the inhibition of Cdc7 produces the greatest difference, the CTL were stimulated for different durations. PHA-767491 treatment did not impair phosphorylation of Lck, ZAP70, LAT, or PLCγ (Figures 7A,B), indicating that Cdc7 inhibition does not influence TCR-proximal signaling. We did detect a transient reduction of Erk phosphorylation in TCR-stimulated cells (Figures 7A,B). To further quantify the extent of the inhibition of Erk phosphorylation, we assessed the level of Erk phosphorylation by flow cytometry. In OT-I CTL that were stimulated with K^b^-OVA tetramers, there is a significant decrease in the percentage of cells with activated Erk (Figure S6A). Similarly, the levels of Erk phosphorylation is impaired in both OT-I peripheral lymphocytes and thymocytes (Figures S6B,C). On the other hand, calcium flux assays indicated that chemical inhibition of Cdc7 does not impair TCR-mediated endoplasmic reticulum calcium release or store-operated calcium entry (SOCE) (Figure 7C). Taken together, our data suggest that Cdc7 is not involved in the regulation of TCR proximal signaling upstream of PLCγ, transiently affects Erk activation, but not the IP_3_-dependent branch of PLCγ signaling.

Stimulation-driven TCR downregulation appears to be impaired in peripheral lymphocytes at early time points, suggesting that the branch of the TCR signaling pathway regulating the receptor ubiquitination and internalization could be affected by the compound (Figure 7D). However, the reverse was observed in thymocytes, where PHA-767491 seems to accentuate TCR internalization in contrast to the vehicle-treated controls (Figure 7D). Moreover, the effect on TCR downregulation is lost in peripheral lymphocytes after prolonged stimulation but is retained in thymocytes (Figure 7D). This effect cannot be directly attributed to known Cdc7 or Cdk9 functions. We therefore sought to test the possibility of direct involvement of one of these kinases, likely Cdc7 (see above), in TCR signaling.

Cdc7 interacts with its cofactor Dbf4, which serves as the regulatory subunit of the Cdc7-Dbf4 binary complex (18, 34). The Cdc7-Dbf4 heterodimer subsequently binds to the MCM helicase and phosphorylates various target sites on the helicase, including the subunit MCM2 (18, 34). As Cdc7 activity is dependent on its association with Dbf4, we wanted to determine if the chemical inhibition of Cdc7 would alter the interaction with Dbf4. Jurkat cells that were cultured overnight in serum-free media were seeded onto anti-CD3 antibody coated plates. The stimulated Jurkat cells were lysed and immunoprecipitated for Dbf4. Our results indicate that TCR stimulation induced the binding of Cdc7 to Dbf4, indicative of Cdc7 activation, which was partially suppressed by PHA-767491-mediated inhibition of Cdc7 (Figure 7E). Significantly, inhibition by PHA-767491 blocked the binding of the Cdc7-Dbf4 complex to MCM2 (Figure 7E). As PHA-767491 is an ATP-competitive inhibitor, it may not significantly impair the association of Cdc7 with Dbf4, but it can be expected to block the kinase activity of Cdc7 and compromise the binding to MCM2, thus inhibiting the phosphorylation of MCM2.

### Effects of PHA-767491 on NF-κB Stability

**Figure 8:**
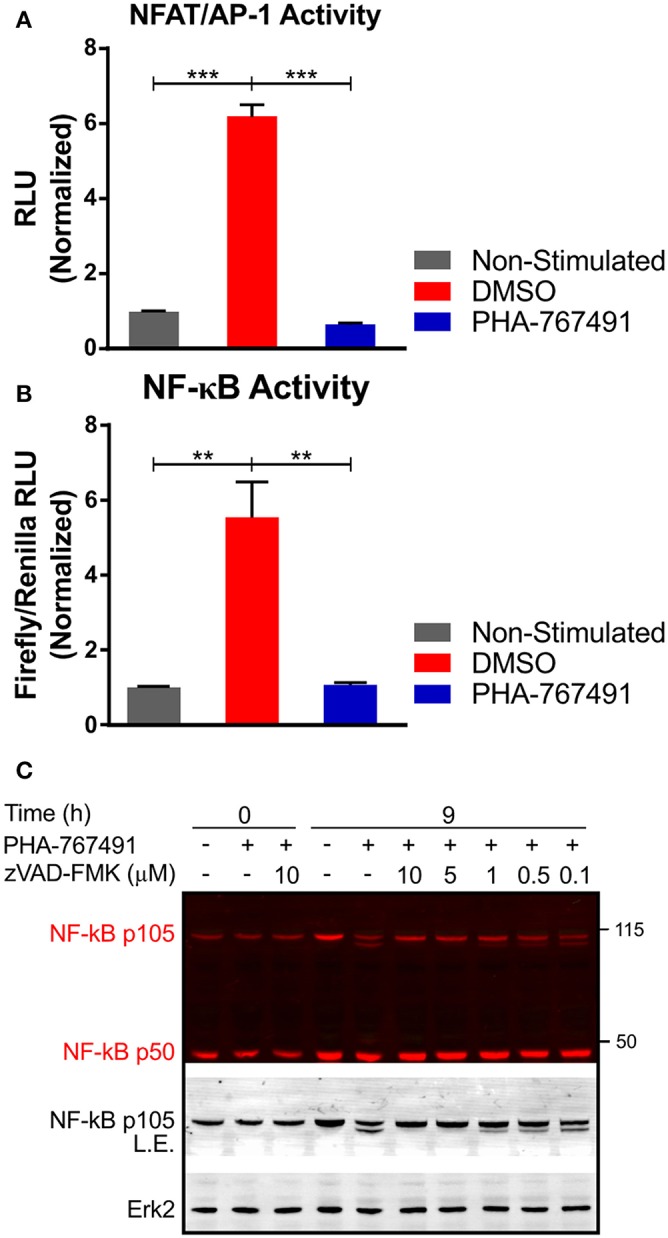
PHA-767491 suppresses activity of transcription factors. **(A)** PHA-767491 impairs the activity of NFAT/AP-1. NFAT/AP-1-luciferase reporter Jurkat cells were treated with DMSO or PHA-767491 and stimulated with plate-bound CD3 antibodies for 6 h. **(B)** PHA-767491 impedes NF-κB activity. NF-κB-firefly luciferase and *Renilla* luciferase control plasmids were electroporated into Jurkat cells. The electroporated cells were cultured in hygromycin selection media before the assay. The NF-κB-firefly luciferase containing Jurkat cells were treated with DMSO or PHA-767491 and stimulated with plate-bound CD3 antibodies for 6 h. NF-κB activity is established as a ratio of the NF-κB-dependent firefly luciferase over the *Renilla* luciferase control. Data shown is representative of at least three independent experiments. Bar charts, represented as mean ± SEM, have been normalized to the NS sample. Statistical significance was determined by unpaired two-sided Student's *t*-test (**p < 0.01; ***p < 0.001). **(C)** Immunoblots of Jurkat cell lysates that were not-treated (–) or treated (+) with PHA-767491 and stimulated with plate-bound anti-CD3 antibodies for 9 h. Samples were either treated with zVAD-FMK at the indicated concentrations or not-treated with the caspase inhibitor. Representative blots of at least three independent experiments are shown.

The transient nature of the effects of Cdc7 inhibition on TCR-proximal signaling prompted us to analyze later events in the TCR signaling cascade. We investigated the activation of transcription factors NFAT and NF-κB which are crucial for various aspects of T cell homeostasis and function (48, 49). PHA-767491 suppressed NFAT/AP-1 activity in Jurkat cells expressing the corresponding luciferase reporter (27) (a kind gift of O. Acuto, Oxford University) stimulated with anti-human CD3 antibodies (Figure 8A). Quantitative studies were also performed for NF-κB activation to confirm NF-κB regulation by Cdc7. We introduced an NF-κB firefly luciferase construct and a *Renilla* luciferase control construct (from O. Acuto, Oxford University) into Jurkat cells. NF-κB activation was assayed after stimulation with anti-human CD3 antibody in the presence or absence of PHA-767491. Similar to NFAT/AP-1, transcriptional activation by NF-κB was strongly inhibited by the drug (Figure 8B).

The lack of NF-κB activity prompted us to look deeper into the NF-κB signaling pathway. PHA-767491 induced an apparent proteolytic cleavage of the p105 isoform of NF-κB. The cleavage resulted in the appearance of a second band of approximately 100 kDa, which retained reactivity with the anti-NF-κB antibody that recognizes the amino terminal of the protein (Figure 8C). The cleavage of NF-κB was likely mediated by active caspase-3 since the application of the specific caspase-3 inhibitor zVAD-FMK suppressed cleavage in a concentration-dependent manner (Figure 8C).

## Discussion

The TCR signaling network is crucial for many aspects of T cell biology, including the development of thymocytes, the activation of T cells, and initiation of their effector functions. Kinases and phosphatases intricately regulate the transduction of signals. In this study, we have identified that a dual inhibitor of Cdc7 and Cdk9, PHA-767491, has suppressive effects on T cell signaling and activation.

As PHA-767491 has two main targets of inhibition, we compared the inhibitory effect of PHA-767491 with other Cdc7 or Cdk9 inhibitors to better understand the underlying mechanisms of PHA-767491 mediated inhibition. Our data reflects the inhibitory strength of the different inhibitors in terms of their suppressive effects on the phosphorylation of the targets of Cdc7 and Cdk9. PHA-767491 inhibited RNAPII phosphorylation more strongly than LDC067, a Cdk9 specific inhibitor, which is reflective of their relative IC50 values—at 34 and 44 nM, respectively (18, 35). XL-413, a Cdc7 inhibitor, inhibited MCM2 phosphorylation more strongly than PHA-767491, which is also mirrored by their IC50 values−10 nM for PHA-767491 and 3.4 nM for XL-413 (18, 19). Another Cdk9 inhibitor, SNS-032, showed high potency in inhibiting T cell activation, even when compared to the other inhibitors. This can be attributed to its ability to inhibit Cdk7, which is important for the initiation of transcription, in addition to Cdk9, which is involved in transcription elongation (23).

We have also observed that PHA-767491 is able to reduce Cdc7 levels. This is in agreement with published results (50) and is likely indicative of either a feedback loop in Cdc7 activation or a progressive loss of Cdc7 due to its transcriptional inactivation. Intriguingly, the same study reports that a combination of Cdc7 inhibition by XL-413 and Cdk9 knockdown failed to reduce Cdc7 levels (50), suggesting that transcriptional suppression is not the mechanism responsible for PHA-767491-mediated downregulation of Cdc7.

The downregulation of Cdc7 is a peculiar observation as the protein levels of Cdc7 do not change between different stages of the cell cycle. Due to the importance of Cdc7 in the regulation of the cell cycle, its activity or expression is expected to be tightly regulated. However, instead of Cdc7, it is the regulatory subunit Dbf4 that exhibits fluctuations of its expressions in tandem with cell cycle progression (8–10). Regulation of Dbf4 expression is sufficient to control Cdc7 activity as its kinase activity is only activated when bound to the Dbf4 regulatory subunit.

The inability of XL-413 to convert its potent molecular inhibition of Cdc7 to long-term cellular readouts, such as the inhibition of proliferation, has previously been reported (33, 50). This is apparent in our assays assessing T cell activation. XL-413 did not show stronger suppression of CD69 or CD25 expression over PHA-767491, which deviates from the earlier observation of the compounds' effect on MCM2 phosphorylation, wherein XL-413 clearly showed a stronger inhibition of MCM2 phosphorylation. Nonetheless, the similarities in the phenotypic effects of both inhibitors suggest that inhibition of Cdc7 can affect T cell activation in a Cdk9-independent manner. However, we cannot rule out the possibility that other targets of XL-413 are responsible for the T cell suppressive effects.

As PHA-767491 inhibits Cdk9, the inhibition of transcription, and hence protein synthesis, can account for several readouts of PHA-767491 mediated suppression of T cell activation. This is evident in the discrepancy in the extent of inhibition on the production of the cytokines TNF and IFN-γ. A transcription-independent mechanism of TNF expression upon stimulation of the TCR (51) could account for a partial suppression of TNF production in spite of transcriptional inhibition.

The suppression of transcription factor activity seen in the luciferase assays can also be attributed to Cdk9 inhibition as the readout was based purely on the luciferase activity. Nonetheless, delving deeper into the effects of PHA-767491 on NF-κB signaling has revealed the influence of caspase-3 on NF-κB stability. Caspase-3 has been shown to be active in stimulated T lymphocytes in a non-apoptotic setting (52), and our results could hint toward the mechanism of how Cdc7 affects T cell signaling.

In our T cell signaling studies, we have shown that PHA-767491 was able to inhibit Erk phosphorylation in different T cell populations, which are in different stages of their cell cycle: with OT-I CTL actively proliferating and peripheral lymphocytes mainly in the G_0_ phase (53). As PHA-767491-mediated inhibition of Erk phosphorylation is reproducible in the different cell populations, this readout is independent of the cell cycle. Critically, the timeframe of the inhibition of early TCR signaling and activation is distinctively different from the timeframe required for entry into the cell cycle.

While we were unable to identify a direct substrate of Cdc7 that mediates the effects of PHA-767491 on T cell signaling and activation, we have shown that Cdc7 activity is inducible upon stimulation. Furthermore, the kinetics of both Erk and MCM2 activation were comparable, occurring within minutes of stimulation, and the phosphorylation of both were inhibited by PHA-767491. Cdc7 is known to be an acidophilic kinase, targeting Ser/Thr residues in sequences with multiple acidic amino acids or sequences with consecutive Ser/Thr residues (54, 55). As Erk possesses multiple Ser/Thr residues in close proximity, Erk could be a possible direct substrate of Cdc7.

There are cell cycle regulating proteins that are involved in the TCR signaling network. Cdc42 is a GTPase that regulates actin dynamics, a process that reorganizes the actin cytoskeleton of the T cell and facilitates the interaction with APCs (56). Recently, another cell cycle protein, Aurora A, has been implicated in TCR signaling (57). The authors showed that Aurora A kinase plays a role in microtubule organization at the immunological synapse and the trafficking and activity of Lck.

In addition to Cdc7's canonical role in the regulation of cell cycle progression, it has also been implicated in several non-canonical roles including: regulation of meiosis, DNA replication checkpoint under replication stress, regulation of histone levels and centromere structural stability, spindle checkpoint and regulation of mitotic exit (58–62). However, these additional roles were studied mainly in budding and fission yeast, and have not been shown in mammals. Nonetheless, there are studies that have shown that Cdc7 is involved in the DNA replication checkpoint in humans: Cdc7 is phosphorylated by the DNA damage checkpoint kinases ATM and ATR to initiate an S phase DNA damage response in the event of exposure to replication stress (63); and Cdc7 indirectly leads to the activation of another DNA damage checkpoint kinase, Chk1, through the phosphorylation of Claspin (64).

The potential role of Cdc7 in influencing centromere integrity and spindle formation is intriguing as it suggests a possible function relating to the cytoskeleton. Our data on the effect of PHA-767491 on TCR downregulation is not a direct consequence of inhibiting the canonical functions of Cdc7 or Cdk9. This phenomenon could be caused by modulating the ubiquitination pathway or, similarly to the other cell cycle proteins mentioned above, an effect on the cytoskeletal dynamics. The role of Aurora A, in addition to our results on Cdc7 and Cdk9, further highlights the role of serine-threonine kinases in the TCR signaling process. Downstream of the TCR, multiple pathways involving serine-threonine kinases, which includes the Ras and Akt signaling pathways, control various events that ensue following T cell activation (65).

To summarize, we provide experimental evidence for a substantial suppression of T cell activation and effector functions upon Cdc7/Cdk9 inhibition. We identified the compound PHA-767491 in a kinase inhibitor screen designed to reveal new molecules affecting T cell development and activation. PHA-767491 potently inhibited the upregulation of surface markers of T cell activation, secretion of most cytokines, and antigen-driven proliferation. Effects of PHA-767491 and other related inhibitors on T cell activation correlate with the inhibition of phosphorylation of their canonical targets, the protein kinases Cdc7 and Cdk9. Importantly, our preliminary data demonstrate that apart from their functions in the regulation of cell cycle progression and gene expression, these kinases may be involved in signal transduction downstream of T cell receptor. For example, we show that although the activation of TCR-proximal signaling machinery is unaffected by PHA-767491, activation of the protein kinase Erk is transiently suppressed, and most intriguingly, the stability of NF-κB p105 is reduced in PHA-767491-treated T cells. Our data show that the application of the compound results in caspase-3-dependent degradation of p105.

Cdc7 and Cdk9 are seen as promising targets in cancer, and multiple compounds targeting these kinases, including PHA-767491, have been investigated for potential anti-cancer effects in preclinical studies and clinical trials. Our data provide evidence that systemic interference with Cdc7 and Cdk9 activities strongly affects T cell responsiveness and may therefore be in fact tolerogenic. In our experiments, T cells were sensitive to Cdc7/Cdk9 inhibition in the range of concentrations similar to those reported for cancer cells. Consequently, although therapeutic inhibition of Cdc7 and Cdk9 continues to provide opportunities in cancer, their impact on the immune system warrants further consideration for clinical application.

## Data Availability

The datasets generated for this study are available on request to the corresponding author.

## Ethics Statement

This study was carried out in accordance with the National University of Singapore IACUC guidelines. The protocol was approved by the National University of Singapore IACUC.

## Author Contributions

NG and VR conceived and supervised the project. EC, NT, JB, and VR designed and performed experiments and analyzed data. EC and VR prepared the initial draft of the manuscript and figures. All authors edited and approved the final version of the manuscript for submission.

### Conflict of Interest Statement

The authors declare that the research was conducted in the absence of any commercial or financial relationships that could be construed as a potential conflict of interest.

## Supplementary Material

Figure S1 Dose response of PHA-767491. **(A)** Dose-response of PHA-767491 on peripheral lymphocytes stimulated with plate-bound anti-CD3 antibody for 24 h. Dose-response curves are plotted separately for both CD4 and CD8 lymphocytes. **(B)** Effect of PHA-767491 titration on the activation of mouse thymocytes and lymphocytes. Thymocytes were stimulated with anti-CD3/CD28 beads and lymphocytes were stimulated with plate-bound anti-CD3 antibodies. The percentages of the positive population of each sample are represented in each graph according to their respective colors. Data shown is representative of at least three independent experiments.

Figure S2 PHA-767491 suppresses MCM2 phosphorylation in cell lines and Cdc7 levels. Immunoblots of the cell lysates from Jurkat, MCF7, and 293T cell lines after treatment with PHA-767491 for the indicated durations. Normalized values of individual bands are indicated below the respective bands. Representative blots of three independent experiments are shown.

Figure S3 MCM2 phosphorylation is a marker of Cdc7 activation at early time points. Immunoblots of cell lysates from Jurkat cells, not treated (–) or treated (+) with PHA-767491, that were stimulated with PMA for the indicated durations. Normalized values of the intensities of the individual bands are indicated below the respective bands. Representative blots of at least three independent experiments are shown.

Figure S4 PHA-767491 inhibits activation of OT-I peripheral T cells. **(A)** Cytokine production in peripheral T cells from both OT-I transgenic and B6 wild-type mice is inhibited by PHA-767491. (Left column) Peripheral lymphocytes from OT-I transgenic mice were pre-treated with BFA for 30 min, treated with DMSO or PHA-767491, and stimulated with K^b^-OVA tetramers for 6 h. (Right columns) Peripheral lymphocytes from B6 wild-type mice were pre-treated with BFA for 30 min, treated with DMSO or PHA-767491, and stimulated with PMA + Ionomycin for 6 h. The percentages of the positive population of each sample are represented in each graph according to their respective colors. **(B)** PHA-767491 suppresses CD69 expression in OT-I peripheral lymphocytes. Peripheral lymphocytes from OT-I transgenic mice were treated with either DMSO or PHA-767491 and stimulated with K^b^-OVA tetramers for 3 h. The percentages of the positive population of each sample are represented in each graph according to their respective colors. **(C)** PHA-767491 inhibits proliferation in OT-I peripheral lymphocytes. Peripheral lymphocytes from OT-I transgenic mice were labeled with CTV, treated with either DMSO or PHA-767491, and were stimulated with K^b^-OVA tetramers for 72 h. The percentages of the proliferating population of each sample are represented in each graph according to their respective colors. Data shown is representative of at least three independent experiments.

Figure S5 Cdc7 inhibitors suppress T cell activation. **(A)** Effect of inhibitors of various cell cycle components on the activation of thymocytes. Thymocytes were stimulated with anti-CD3/CD28 beads for 17 h. Graphs, shown as mean ± SEM, compare the percentage of active caspase-3 and CD69 expressing cells for PHA767491-treated samples to the assay controls and other inhibitors. **(B,C)** Chemical inhibitors of Cdc7 impair T cell activation. Peripheral lymphocytes were stimulated with plate-bound anti-CD3 antibody for 3 h. Histograms depict the effect of the Cdc7 inhibitors on **(B)** CD69 expression and TCR downregulation and **(C)** the dose-response of PHA-767491 and XL-413 treatment on CD69 expression. The percentages of the positive population of each sample are represented in each graph according to their respective colors. Data shown is representative of at least three independent experiments.

Figure S6 PHA-767491 suppresses Erk phosphorylation. PHA-767491 impairs the phosphorylation of Erk in **(A)** OT-I CTL, **(B)** OT-I peripheral lymphocytes, and **(C)** OT-I thymocytes. The cells were treated with either DMSO or PHA-767491 and stimulated with K^b^-OVA tetramers for 60 s. PMA was used as a positive control for Erk phosphorylation. The percentages of the positive population of each sample are represented in each graph according to their respective colors. Data shown is representative of at least three independent experiments. Bar charts, represented as mean ± SEM, have been normalized to the NS sample. Statistical significance was determined by unpaired two-sided Student's *t*-test (^*^*p* < 0.05; ^***^*p* < 0.001).
