# Supplementary figures and images for: A Dual Inhibitor of Cdc7/Cdk9 Potently Suppresses T Cell Activation
Source: Front Immunol. 2019 Jul 25;10:1718. doi: 10.3389/fimmu.2019.01718 (PMC6670834; doi:10.3389/fimmu.2019.01718)

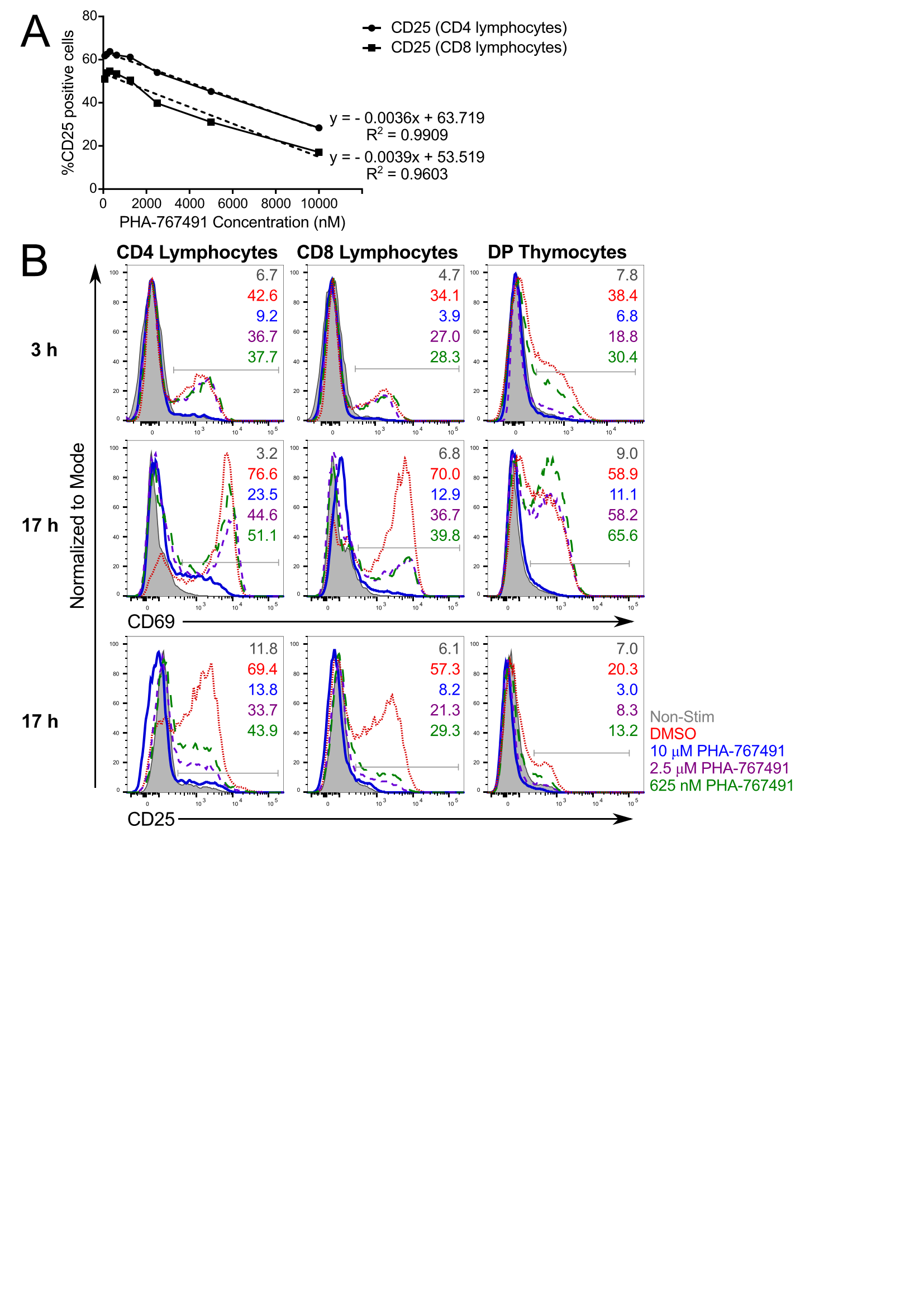

Supplement: Figure S1 — Dose response of PHA-767491. (A) Dose-response of PHA-767491 on peripheral lymphocytes stimulated with plate-bound anti-CD3 antibody for 24 h. Dose-response curves are plotted separately for both CD4 and CD8 lymphocytes. (B) Effect of PHA-767491 titration on the activation of mouse thymocytes and lymphocytes. Thymocytes were stimulated with anti-CD3/CD28 beads and lymphocytes were stimulated with plate-bound anti-CD3 antibodies. The percentages of the positive population of each sample are represented in each graph according to their respective colors. Data shown is representative of at least three independent experiments. [file Image_1.TIFF]

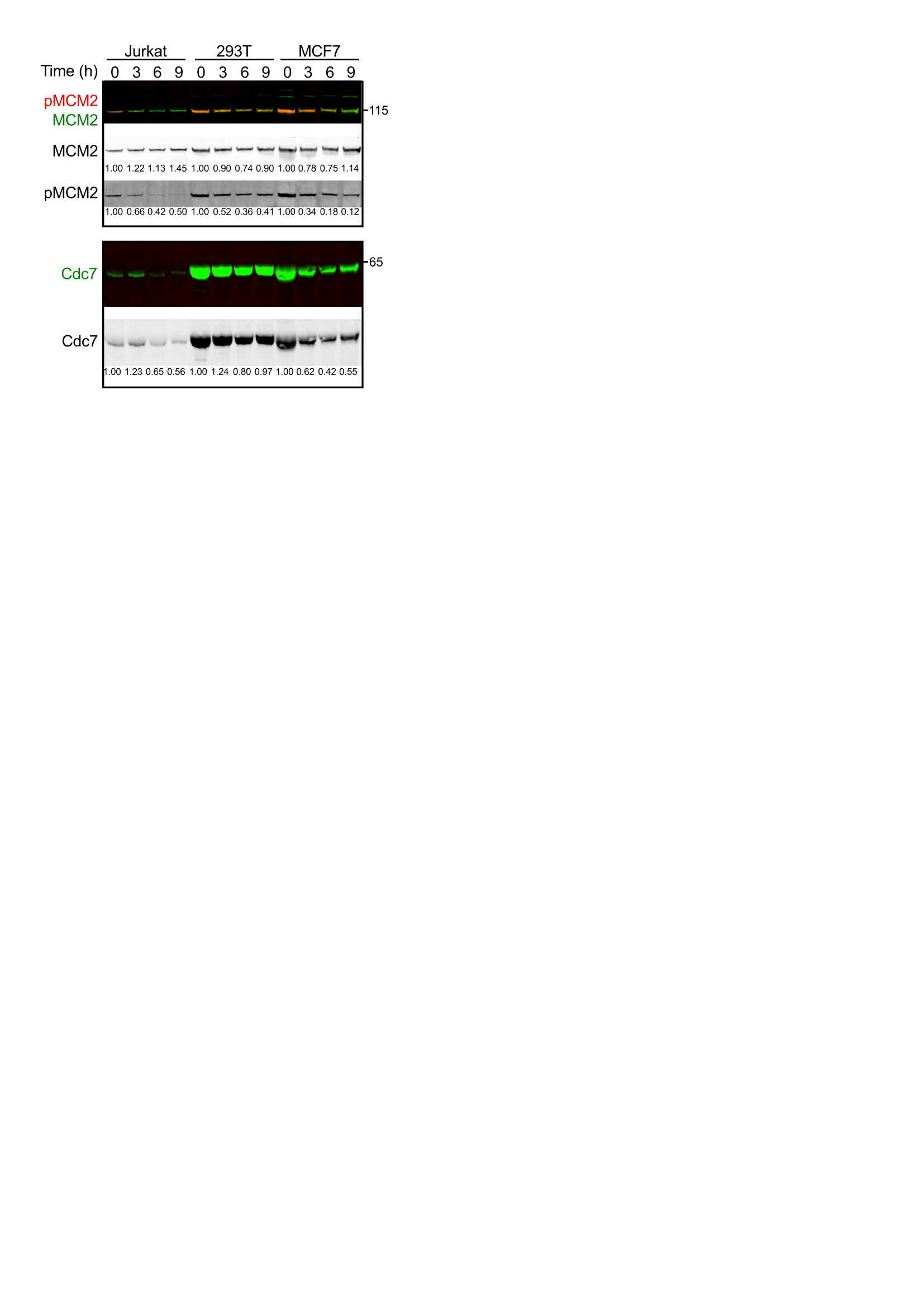

Supplement: Figure S2 — PHA-767491 suppresses MCM2 phosphorylation in cell lines and Cdc7 levels. Immunoblots of the cell lysates from Jurkat, MCF7, and 293T cell lines after treatment with PHA-767491 for the indicated durations. Normalized values of individual bands are indicated below the respective bands. Representative blots of three independent experiments are shown. [file Image_2.TIFF]

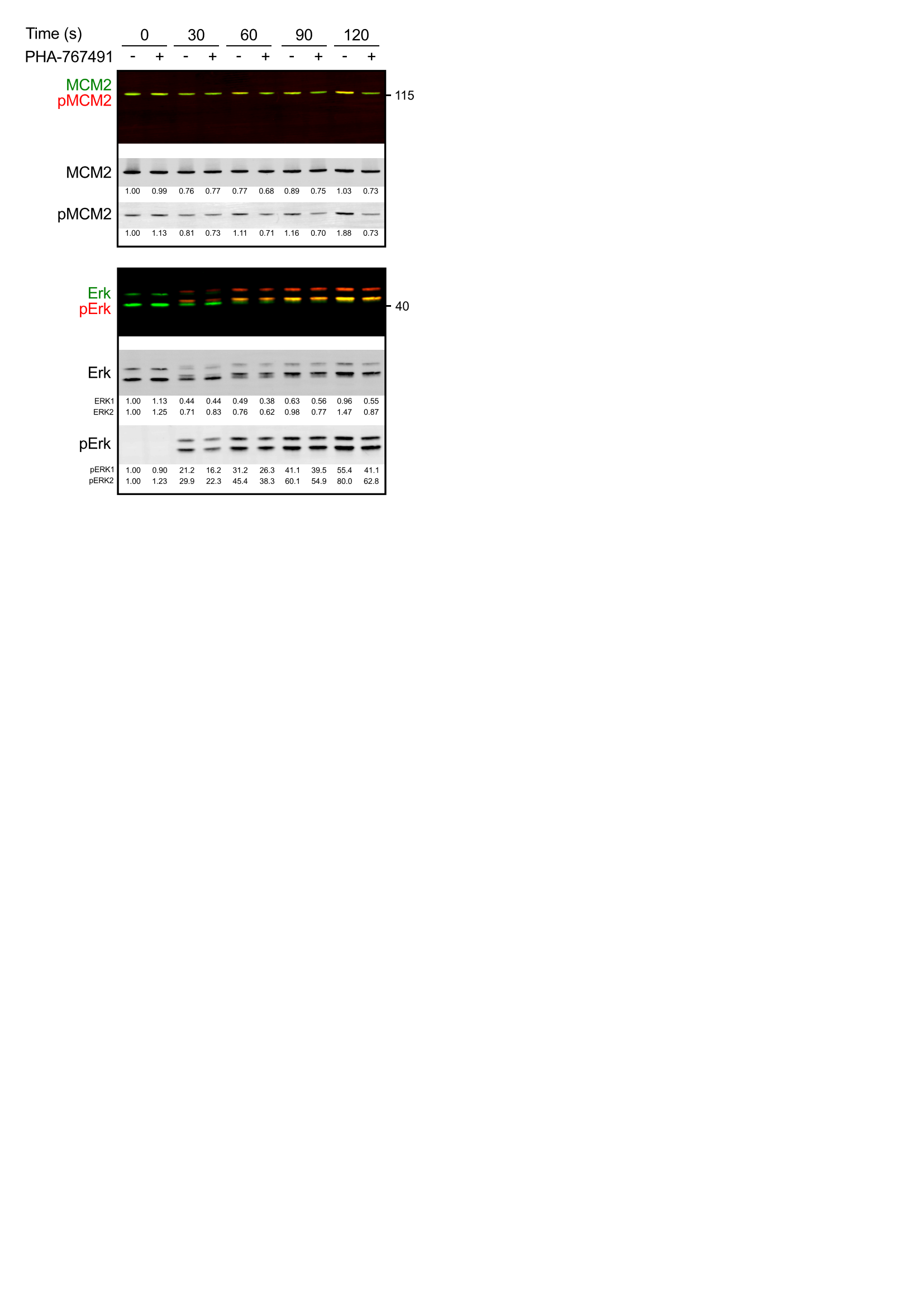

Supplement: Figure S3 — MCM2 phosphorylation is a marker of Cdc7 activation at early time points. Immunoblots of cell lysates from Jurkat cells, not treated (–) or treated (+) with PHA-767491, that were stimulated with PMA for the indicated durations. Normalized values of the intensities of the individual bands are indicated below the respective bands. Representative blots of at least three independent experiments are shown. [file Image_3.TIFF]

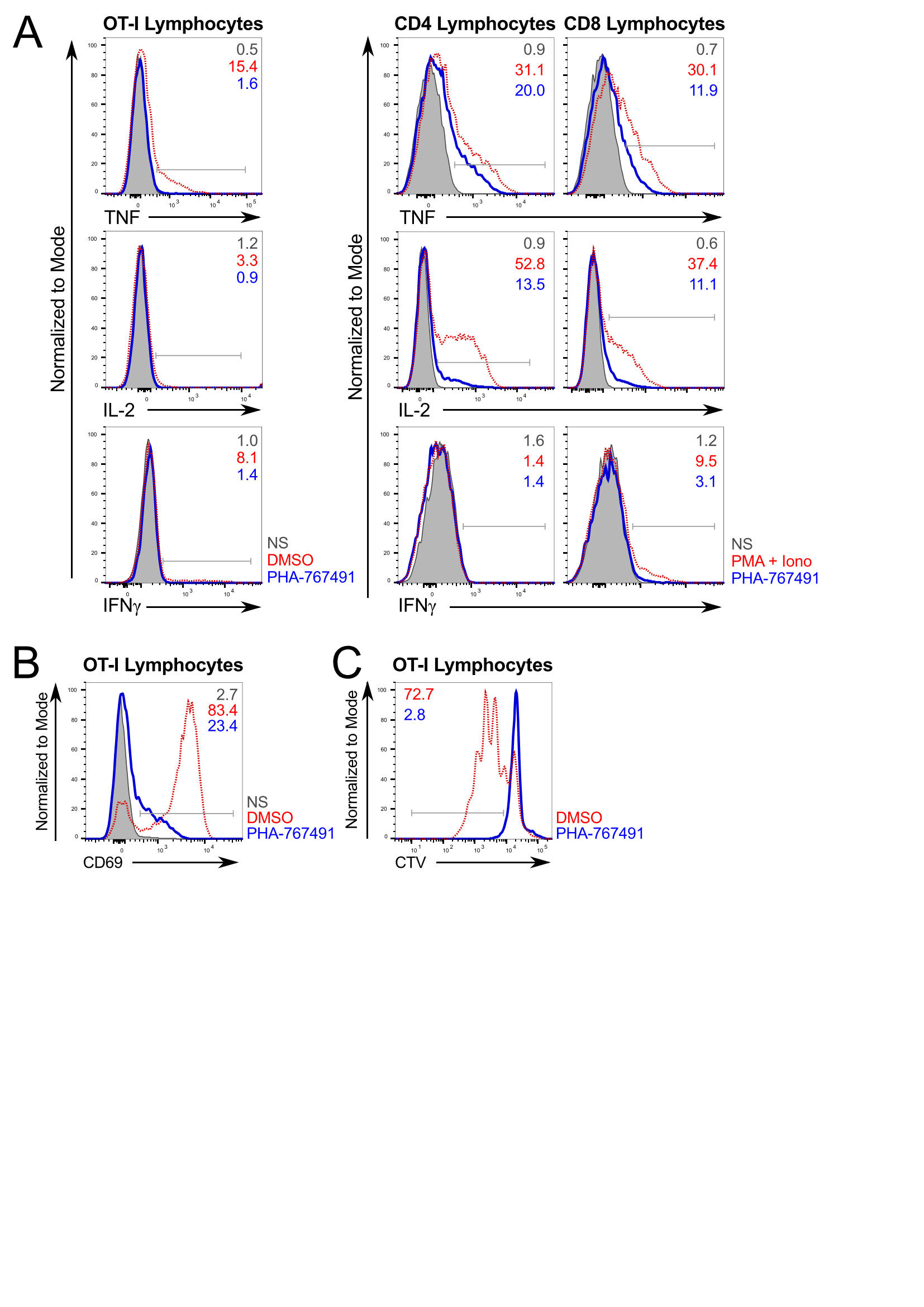

Supplement: Figure S4 — PHA-767491 inhibits activation of OT-I peripheral T cells. (A) Cytokine production in peripheral T cells from both OT-I transgenic and B6 wild-type mice is inhibited by PHA-767491. (Left column) Peripheral lymphocytes from OT-I transgenic mice were pre-treated with BFA for 30 min, treated with DMSO or PHA-767491, and stimulated with Kb-OVA tetramers for 6 h. (Right columns) Peripheral lymphocytes from B6 wild-type mice were pre-treated with BFA for 30 min, treated with DMSO or PHA-767491, and stimulated with PMA + Ionomycin for 6 h. The percentages of the positive population of each sample are represented in each graph according to their respective colors. (B) PHA-767491 suppresses CD69 expression in OT-I peripheral lymphocytes. Peripheral lymphocytes from OT-I transgenic mice were treated with either DMSO or PHA-767491 and stimulated with Kb-OVA tetramers for 3 h. The percentages of the positive population of each sample are represented in each graph according to their respective colors. (C) PHA-767491 inhibits proliferation in OT-I peripheral lymphocytes. Peripheral lymphocytes from OT-I transgenic mice were labeled with CTV, treated with either DMSO or PHA-767491, and were stimulated with Kb-OVA tetramers for 72 h. The percentages of the proliferating population of each sample are represented in each graph according to their respective colors. Data shown is representative of at least three independent experiments. [file Image_4.TIFF]

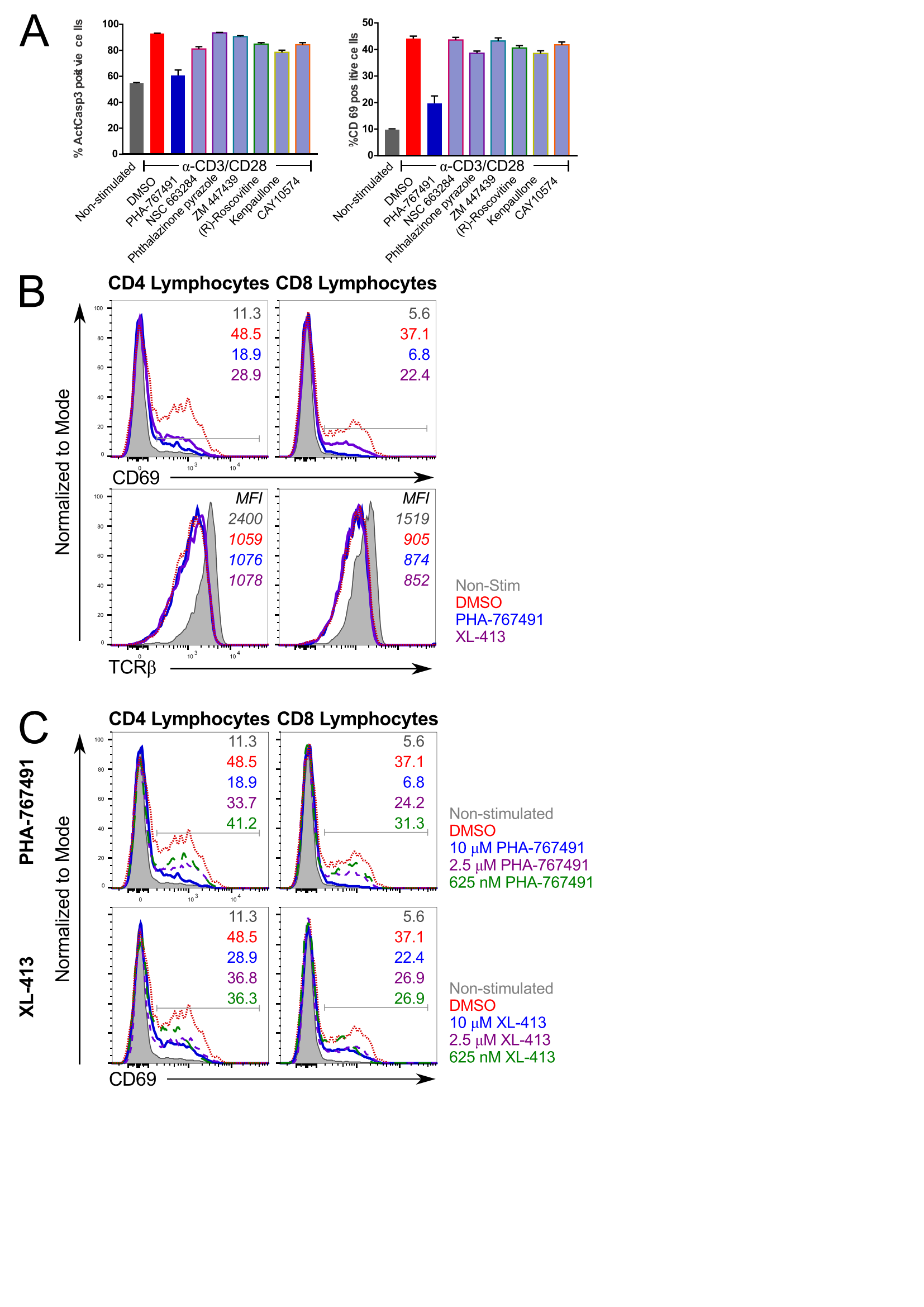

Supplement: Figure S5 — Cdc7 inhibitors suppress T cell activation. (A) Effect of inhibitors of various cell cycle components on the activation of thymocytes. Thymocytes were stimulated with anti-CD3/CD28 beads for 17 h. Graphs, shown as mean ± SEM, compare the percentage of active caspase-3 and CD69 expressing cells for PHA767491-treated samples to the assay controls and other inhibitors. (B,C) Chemical inhibitors of Cdc7 impair T cell activation. Peripheral lymphocytes were stimulated with plate-bound anti-CD3 antibody for 3 h. Histograms depict the effect of the Cdc7 inhibitors on (B) CD69 expression and TCR downregulation and (C) the dose-response of PHA-767491 and XL-413 treatment on CD69 expression. The percentages of the positive population of each sample are represented in each graph according to their respective colors. Data shown is representative of at least three independent experiments. [file Image_5.TIFF]

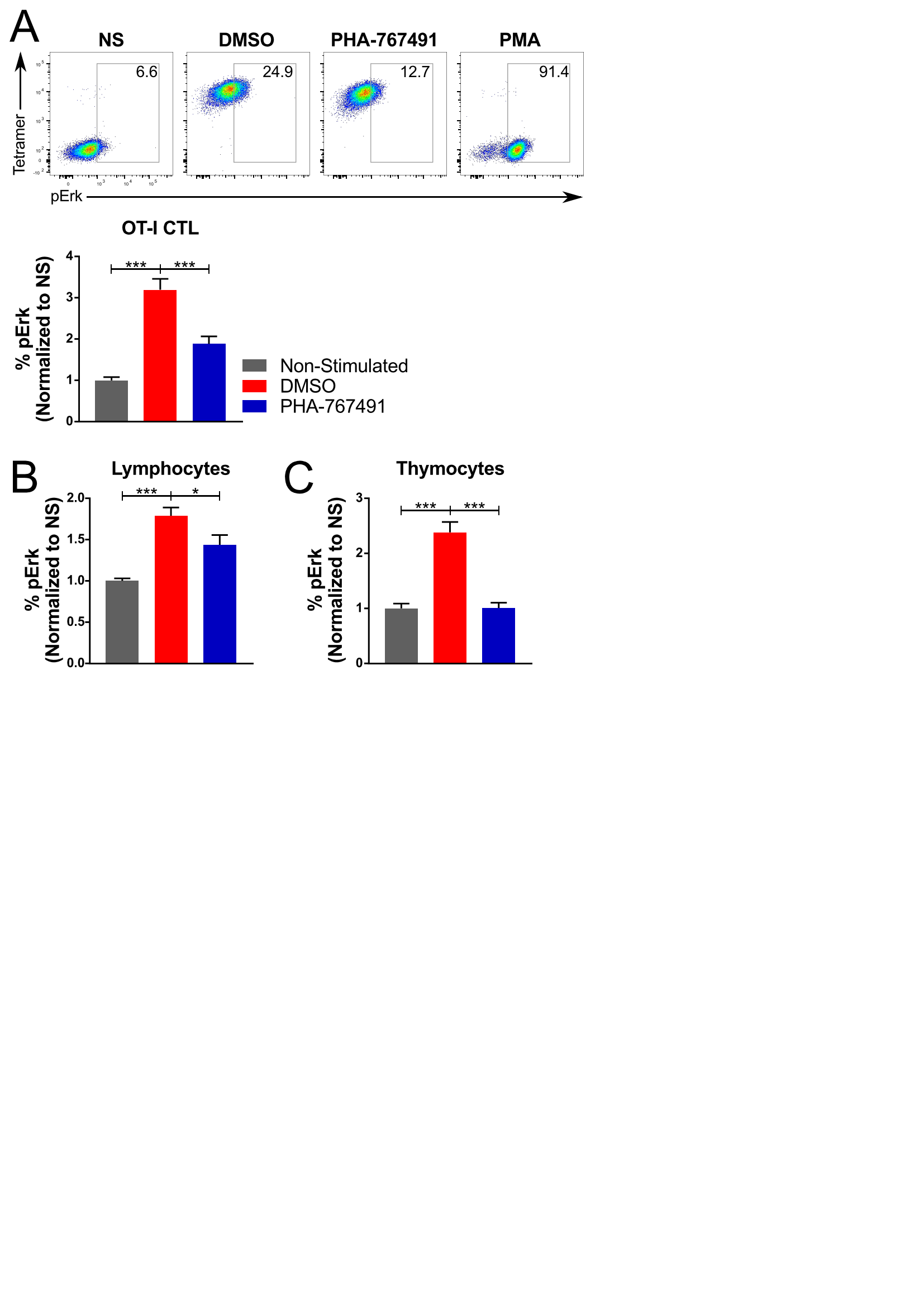

Supplement: Figure S6 — PHA-767491 suppresses Erk phosphorylation. PHA-767491 impairs the phosphorylation of Erk in (A) OT-I CTL, (B) OT-I peripheral lymphocytes, and (C) OT-I thymocytes. The cells were treated with either DMSO or PHA-767491 and stimulated with Kb-OVA tetramers for 60 s. PMA was used as a positive control for Erk phosphorylation. The percentages of the positive population of each sample are represented in each graph according to their respective colors. Data shown is representative of at least three independent experiments. Bar charts, represented as mean ± SEM, have been normalized to the NS sample. Statistical significance was determined by unpaired two-sided Student's t-test (*p < 0.05; ***p < 0.001). [file Image_6.TIFF]
